# Evidence-Based Strategies for Improving Diversity and Inclusion in Undergraduate Research Labs

**DOI:** 10.3389/fpsyg.2019.01305

**Published:** 2019-06-18

**Authors:** Afra Saeed Ahmad, Isaac Sabat, Rachel Trump-Steele, Eden King

**Affiliations:** ^1^Department of Psychology, George Mason University, Fairfax, VA, United States; ^2^Department of Psychology, Texas A&M University, College Station, TX, United States; ^3^Department of Psychological Sciences, Rice University, Houston, TX, United States

**Keywords:** diversity, inclusion, undergraduate students, research assistants, faculty mentors

Institutions of higher education strive to support diversity and inclusion efforts as they recognize the benefits at the undergraduate, graduate, and faculty levels (Terenzini et al., 2001; Denson and Chang, 2009; Pascarella et al., 2014; Moriña, 2017). Diversity can be defined as “the varied perspectives and approaches to work which members of different identity groups bring” (Thomas and Ely, 1996) and inclusion can be described as a person's ability to contribute fully and effectively to an organization (Miller, 1998; Mor Barak and Cherin, 1998). One strategy to diversify higher education is by focusing on creating a diverse pipeline, whereby undergraduates from different backgrounds engage in high quality research. These experiences provide students the ability to build competencies and achievement records that propel them to and through graduate school as well as beyond.

Previous research has demonstrated that undergraduates who participate in research projects and positively interact with faculty are more likely to pursue and attain post-baccalaureate degrees as well as subsequent careers as faculty or research scientists (Pascarella and Terenzini, 1991; Astin, 1993; Tinto, 1993; Adedokun et al., 2013; Yaffe et al., 2014). Opportunity and mentorship are particularly critical for underrepresented students, as previous research has found that students' interactions with faculty members have a stronger influence on their decisions to pursue graduate education than their initial background characteristics (e.g., socio-economic status; Ethington and Smart, 1986; Pascarella and Terenzini, 1991; Carpi et al., 2017). While many mentors may intend to support minority student researchers, they may not be aware of how to do so. Thus, this paper will highlight some of the challenges faced by underrepresented students (i.e., students of color, lower socio-economic status, LGBT) and provide evidence-based solutions on how to recruit, select, retain students from diverse backgrounds to promote diversity and inclusion in undergraduate research labs working toward publishable research.

## Promoting an Inclusive Research Lab

Multiple studies have found that minority students report feeling isolated, unwelcomed, invisible and distant from faculty (Fullilove and Treisman, 1990; Rankin, 2003; Suarez-Balcazar et al., 2003; Love, 2008; Cherng et al., 2014). Inclusive research lab practices related to recruiting, selecting and retaining diverse student researchers can reduce the effects of these negative experiences.

### Recruitment

As recruitment may be one of the first barriers faced in achieving a diverse research lab, active recruitment efforts must complement other efforts to get diverse students in the door. Active recruitment is defined as efforts that may aid in an increase in applicants and have been used to attract minority applicants to different graduate program and professions (George et al., 1997; Muñoz-Dunbar and Stanton, 1999). Researchers in organizational psychology have found that more diverse recruitment advertisements positively impact perceptions of organizational attractiveness, perceived compatibility, and evaluations of the organization's image (Perkins et al., 2000; Avery et al., 2004; Lambert, 2015; Baum et al., 2016). Based on these findings, we suggest that advertisements for student research opportunities should include pictures of diverse students and explicit statements encouraging students of all backgrounds to apply. Additionally, it is important for recruitment advertisements to use language that can directly combat some of the misperceptions about research labs that may persuade students from various backgrounds to select-out of participating, as they feel that they may not fit in. For example, students from lower socio-economic status backgrounds may be juggling both work and academic demands and feel that they are not able to participate in research. However, recruitment messages may be tailored to address this need by mentioning the option of flexibility in hours and location for work to be conducted, if applicable. These recruitment efforts may help to attract students from all backgrounds who typically feel excluded from these opportunities as they signal inclusiveness through pictures and messages.

Proactive types of recruitment efforts can take place by both faculty and lab members. For example, faculty members can engaging in mentoring behaviors during the recruitment process. This can be done by faculty identifying and encouraging strong minority students in the classroom to apply for research opportunities. Oftentimes, students from underrepresented groups are anxious and feel they do not belong due to a lack of representation. Previous work in educational psychology found that high school students express self-doubt based on an unwelcoming culture of seeing an all-white AP classroom, even after being accepted to these challenging courses (Belcher, 2017). However, with further encouragement and longer discussion from mentors, 90% of those who opted not to originally take AP courses did eventually do so. Similarly, it is likely that minority students doubt their abilities to work in high quality research labs, but may overcome this barrier with appropriate mentorship.

Current lab members may also take an active role in recruitment diverse students. For example, research assistants can set time aside for community outreach events where diverse students may be involved in, such as sports, student clubs, or special events on campus. This can allow for the opportunity for current students to engage in conversation about their experiences working in a lab and the benefits of research for their future goals, specifically, articulating that they are working on publishable research which will be instrumental for pursuing graduate education.

### Selection

Biases can negatively influence minority students' experiences in being provided opportunities in a research lab. Research has found that minority students report experiences of discrimination and differential treatment from their advisors and from prospective advisors (Rankin, 2003; Suarez-Balcazar et al., 2003; Shammas, 2017). For example, faculty were more responsive to White male undergraduate students when contacted about prospective research and mentorship opportunities compared to female or ethnically diverse students (Milkman et al., 2015). Additionally, faculty members rated male lab manager applicants (identical to female counterparts) as more competent and hireable, as well as deserving of a higher salary and more mentorship (Moss-Racusin et al., 2012). Biases are held by all, and faculty who consider themselves free from bias (Staats et al., 2015) or who share these minority characteristics are not immune (Durso and Latner, 2008; Herek et al., 2009, 2015).

In light of these findings, it is important for mentors to actively strive to minimize the influence of unintentional bias. One way to determine whether selection processes are impacted by these biases is to conduct regular audits of one's lab to ensure that certain types of students are not systematically being evaluated more poorly than others (Tetlock and Mitchell, 2009). To improve the fairness and accuracy of evaluations, faculty need to set clear, objective, behavior-based performance metrics. Indeed, more general and subjective ratings allow for a greater reliance on these subtle biases (Prendergast and Topel, 1993; Aranda et al., 2014). Research has also demonstrated the importance of learning about implicit biases and taking efforts to recognize and reduce behavioral manifestations of such biases. Ignoring implicit biases will negatively impact the validity of faculty's selection and evaluation systems preventing diverse students from working on impactful research.

Faculty members, especially at larger institutions, often develop some type of selection system for their research labs to engage students to support publishable projects. This process often involves using different sources of information, including SAT scores. However, standardized testing generally disadvantages marginalized applicants (Roth et al., 2001; Dean et al., 2008; Fagioli, 2013) due to several reasons including economic and socioeconomic factors, psychological factors, societal factors, cultural factors, test constructruction, and valdiation factors (Ployhart et al., 2003; Berry et al., 2011). As a consequence, organizational psychologists encourage decision makers in the workplace to broaden perspectives on selection in general. McKay and Davis (2008) argued that in addition to relying on valid, standardized selection instruments, organizations must “expand the number of predictor constructs measured by selection systems beyond cognitive-based tests” (p. 152). They further argue that, “personnel practitioners should include non-cognitive constructs in selection systems to complement organizations' diversity efforts” (p. 153). Following the model of workplace selection practices and extending them to selecting undergraduate researchers, faculty should conduct a job analysis to identify the responsibilities and qualifications that are necessary to be successful in working on publishable research projects and consider alternative ways to assess these skills.

For example, additional criteria that can be used to evaluate student researchers may include factors, such as motivation and research interests. The Council on Graduate Medical Education found motivation to commit time and effort to studying in high demanding programs to be a predictor of success among medical students from minority groups (Pacquiao, 2007). Additionally, we recommend incorporating qualitative approaches to elicit this information by asking students to write a short essay describing their reasons for wanting to join a specific lab, their future career plans and research interests. Educational psychologists have found the essay approach to be useful in assessing underrepresented students' motivation for advanced placement (AP) courses in high school (Belcher, 2017).

### Retention

To retain and support diverse students after the recruitment and selection process, it is important for faculty to engage in mentorship, ally behaviors and encourage diversity more broadly to promote an inclusive lab environment.

#### Mentorship

Students from diverse backgrounds report that a lack of mentorship is a challenge in navigating their educational experiences. Previous work has found that mentoring can be particularly vital to maintaining persistence toward a degree for African American students (Freeman, 1999; Dodson et al., 2009; Blackwell and Pinder, 2014). Notably, African-American students reported higher satisfaction with research-focused faculty support than other types of mentoring (Ishiyama, 2007; Strayhorn and Saddler, 2009; Kendricks et al., 2013; Castellanos et al., 2016).

Therefore, once students are in the lab, faculty members can take an active role in mentorship by providing developmental opportunities (i.e., co-authorship for publications or conferences) and feedback on research related tasks to build the skills of these students at the undergraduate level. These mentorship relationships between diverse students and faculty can foster research publications as well. A research study found that faculty members who had mentored Black or students with disabilities were more productive in publishing with their undergraduates (Morales et al., 2017). The authors suggest that research publication success is likely due to a mentor's commitment given that often additional time and support is needed for socially marginalized students (Sax et al., 2002; Eagan and Garvey, 2015) and team diversity contributing to broader knowledge and skills (Barjak and Robinson, 2008).

Mentors can also provide support for students working toward graduate degrees. The work of several researchers suggests that providing graduate applicants with guidance on what is being sought in professional statements, how to approach letter writers and what to share with them, and how decisions are made can help put those with less experience in higher education (e.g., first generation college students or graduate students, persons from under-represented groups) in a better position to pursue graduate studies (McKay and Davis, 2008; Sedlacek, 2017; Mathur et al., 2019). This process provides everyone with required information and support, creating a more level playing field for pursuing graduate education that otherwise might only be accessible to some. Overall, faculty mentorship enables the process of engaging diverse students in publishable research and beyond.

#### Promote Ally Behaviors

Allies can be both faculty or other lab mentors with similar characteristics and background as diverse students (i.e., faculty of color) or from a majority group (i.e., White student). Faculty can use their own positions of privilege to be allies and model these behaviors for all lab members. To be effective allies, lab mentors can educate themselves on the various barriers faced by each group as well as the strategies that are most effective at supporting and advocating for these groups (Sabat et al., 2013). This can be done by attending ally and other optional diversity training programs, reading, and staying current on research pertaining to organizational diversity and discrimination, participating in diversity-related events, examining one's own biases, and the ways in which they may be perpetuating systematic inequalities, and by developing and fostering diverse social support networks. Using this knowledge, mentors can engage in behaviors that outwardly support diversity by proactively expressing their ally identities and by role modeling their support for all diverse groups. Specifically, they can emphatically state their genuine support for minority groups and diversity-supportive causes, advertise diversity-related events on campus, and post-ally/diversity-supportive stickers in their offices. Individuals who express their ally identities in these ways are likely to create safe spaces.

#### Promoting an Inclusive Lab

The positive environment cultivated in the research lab will likely support both minority and majority students. Mentors engaging and promoting inclusive behaviors may encourage students with concealable stigmas feel comfortable disclosing their identities within the research lab (Sabat et al., 2017). This will likely have a positive impact on marginalized research assistants as disclosure of more hidden identities (i.e., sexual orientation) has been consistently linked to improved satisfaction, commitment, and workplace health (Sabat et al., 2017). Additionally, mentors who disclose their ally identities are also likely to encourage majority members within the lab to feel comfortable acknowledging or disclosing their own ally identities, which will continue to spur a cycle of support allowing all students to thrive in the research lab.

Research has demonstrated that celebrating diversity and taking a multicultural, identity-affirming approach is more beneficial than taking a color-blind approach in which one ignores identity-based differences (Meeussen et al., 2014). Diversity likely already exists in all labs when considering an intersectional framework. Engaging in discussions regarding gender diversity, socio-economic status, sexual orientation, rural/urban upbringing, religious variations, and then seeking to diversify in specific ways (e.g., ethnic diversity) can help all members feel included in diversity related initiatives.

Diversity impacts all aspects of one's experiences and denying this perpetuates systematic disadvantages faced by minority groups (Purdie-Vaughns et al., 2008; Fryberg and Stephens, 2010; Offermann et al., 2014; Bonilla-Silva, 2015). For these reasons, as issues pertaining to diversity arise in the local or national contexts, faculty can allow their lab to be a safe place to address them. Previous evidence has found that broader diversity issues can impact the motivation, well-being, and performance of students, particularly those who are underrepresented (Cokley, 2000; Pugh et al., 2008; Sliter et al., 2014; Prewitt, 2015). Consequently, engaging in these potentially challenging conversations in the lab can help to foster inclusion, model civil conversation, and allow the opportunity for diverse perspectives to be shared. These inclusive practices related to recruitment, selection, and lab management can allow diverse students to feel supported working on high quality research.

## Conclusion

In this paper, we have identified some ways to overcome the challenges faced by underrepresented students including experiences of bias, feelings of isolation, and a lack of mentorship. We have offered solutions to overcome these challenges with regards to recruiting, selecting, and retaining diverse undergraduate researchers working toward publishable work. As the country diversifies and the education system broadens to include online learning, all types of opportunities, including participation in research labs should be accessible to everyone. Together, with our collective efforts, we can move toward more equitable educational institutions that can lead the way in providing equal educational opportunities to all. As noted by the Dean of Harvard College at commencement, “Diversity in the student body is important for the same reason that it is important in research. It is the only way to advance a field… through a diversity of perspectives” (Powell, 2018).

## Author Contributions

AA, IS, RT-S, and EK worked together to create an outline for the manuscript. RT-S identified challenges. AS highlighted recruitment and selection efforts. IS worked on managing labs. All authors worked on revising the manuscript.

### Conflict of Interest Statement

The authors declare that the research was conducted in the absence of any commercial or financial relationships that could be construed as a potential conflict of interest.
